# DNA Repair Defect and *RAS* Mutation in Two Patients With *Schistosoma mansoni*–Associated Colorectal Cancer: Carcinogenesis Steps or Mere Coincidence?

**DOI:** 10.1200/JGO.2016.006254

**Published:** 2016-08-24

**Authors:** Gustavo Fernandes Godoy Almeida, Filipe Wanick Sarinho, Paula Carvalho de Abreu e Lima, Joao Bosco Oliveira Filho, Maxwell Alex de Lima Moura, Lais Neares Barbosa Ribeiro, Bruno Rolim de Brito, Mariana Montenegro de Melo Lira, Marcelo do Rego Maciel Souto Maior, Ana Lucia Coutinho Domingues

**Affiliations:** **Gustavo Fernandes Godoy Almeida**, **Filipe Wanick Sarinho**, **Bruno Rolim de Brito**, **Mariana Montenegro de Melo Lira**, **Marcelo do Rego Maciel Souto Maior**, and **Ana Lucia Coutinho Domingues**, Hospital das Clinicas Federal University of Pernambuco; **Paula Carvalho de Abreu e Lima**, Laboratorio de Patologia Adonis Carvalho; **Joao Bosco Oliveira Filho**, Genomika Diagnosticos; and **Maxwell Alex de Lima Moura** and **Lais Neares Barbosa Ribeiro**, Federal University of Pernambuco, Recife, Brazil.

## INTRODUCTION

Schistosomiasis is caused by nematode worms of the *Schistosoma* genus, including *Schistosoma mansoni*, *Schistosoma japonicum*, and *Schistosoma haematobium* as the main species. It is an endemic disease in tropical and subtropical regions.^1^ At least 230 million people worldwide are infested with *Schistosoma* species.^2^ In Brazil, approximately 25 million people live in areas at risk for *S mansoni*.^3^

*Schistosoma* eggs accumulate in the submucosa of the colon and induce inflammation, which triggers a severe granulomatous reaction that is complicated by microabscesses, ulceration, nodules, polyps, and hyperplasia.^4^ Along with hyperplasia, it has been observed that *S japonicum* eggs induce colorectal carcinoma (CRC).^5,6^

Besides CRC, *S japonicum* has also been implicated in liver cancer development.^4^ In addition, an association between *S haematobium* and bladder cancer has also been described.^7^ However, the association between *S mansoni* and CRC is scarce in the literature. In patients with *S mansoni*–associated CRC, patients are younger, their tumors are multicentric and present with mucinous histology, and there is a greater risk of lymph node metastasis and microsatellite instability (MSI).^8^ We report two patients with concurrent diagnosis of CRC and intestinal schistosomiasis and the potentially implicated carcinogenesis steps.

## CASE REPORTS

The first patient was a 45-year-old woman who presented with abdominal pain, weight loss, and diarrhea. She underwent a colonoscopy in October 2014, which revealed a 3-cm tumor in her cecum. A right colectomy was performed in January 2015, and a well-differentiated mucinous adenocarcinoma of 2.5 × 1.5 × 1.5 cm invading into the muscularis propria was identified. No perineural or lymphovascular invasion was observed, but a mild tumor inflammatory infiltrate was present. Margins were free, and metastasis to one of 24 lymph nodes was documented. Ileal schistosomiasis was found in the specimen. MSI was confirmed by immunohistochemistry (loss of MLH1 and PMS2). All *RAS* mutations were negative. She received 6-month adjuvant capecitabine- and oxaliplatin-based chemotherapy. Last follow-up visit was on June 13, 2016.

The second patient was a 47-year-old man who had a personal history of hepatosplenic schistosomiasis. In 2012, he underwent a right hemicolectomy as a result of complications of appendicitis. In March 2014, splenectomy and an esophageal varices clamp were performed as a result of GI hemorrhage. In November 2014, he presented with diarrhea, and colonoscopy showed a 2-cm tumor next to the ileum–transverse colon anastomosis. In March 2015, the specimen analyzed from a segmental colectomy showed a 3.5 × 1.8 cm mucinous moderately differentiated adenocarcinoma infiltrating subserosa, with free margins, presence of lymphovascular invasion, no perineural infiltration, and a mild lymphocytic infiltrate observed. No lymph nodes were identified in the specimen, but a granulomatous reaction in response to *Schistosoma* eggs in his ileum and colonic mucosa and Merkel diverticula were described by the pathologist. MSI was negative by immunohistochemistry, but exon 2 *KRAS* mutation (c.38G>A:p.G13D) was identified. Because of his comorbidities, he did not receive adjuvant chemotherapy. Last follow-up visit was on June 13, 2016.

In both patients, *KRAS*/*NRAS* exons 2, 3, and 4 were amplified by polymerase chain reaction, and second-generation sequencing was performed using MiSeq (Illumina, San Diego, CA). The patients were tested for MSI using the immunohistochemistry antibodies MLH1, MSH2, MSH6, and PMS2.

## DISCUSSION

Whether *Schistosoma* induces carcinogenesis and its steps is not clear yet. Hanahan and Weinberg^10^ have proposed six hallmarks of cancer that they define as “distinctive and complementary capabilities that enable tumor growth and metastatic dissemination.” These include sustained proliferative signaling, evading growth suppressors, resisting cell death, enabling replicative immortality, inducing angiogenesis, and activating invasion and metastasis. In addition to these six hallmarks, Hanahan and Weinberg^10^ outline two emerging hallmarks and two enabling characteristics that make it possible for tumor cells to acquire the core hallmarks. The two emerging hallmarks are deregulating cellular energetics and avoiding immune destruction. The two enabling characteristics are properties of cancer cells that facilitate the acquisition of the hallmarks. The first of these characteristics is genomic instability, which enables the acquisition of the multiple mutations required for multistep tumorigenesis. The second enabling characteristic is tumor-promoting inflammation, which reflects the rapidly advancing concept that inflammatory responses can actually facilitate tumor initiation and progression in some contexts.^10^ According to these hallmarks, we found in the literature some evidence of the carcinogenesis steps involving schistosomiasis (Table 1).

**Table 1:**
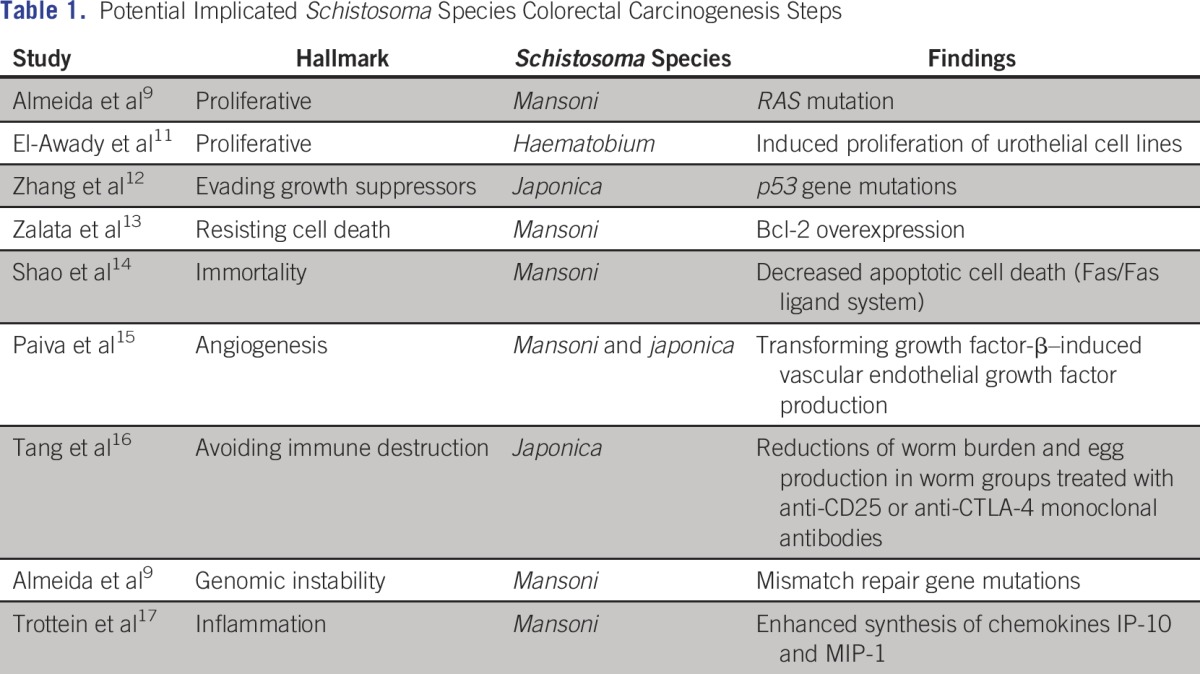
– Potential Implicated *Schistosoma* Species Colorectal Carcinogenesis Steps

In conclusion, the age of the patients and their mucinous subtype were in accordance with the literature.^8^ *RAS* mutation, along with the presence of MSI, may be implicated in the carcinogenesis of *S mansoni*–associated CRC or represent coincidental events. If the first is correct, it would determine treatment and prognosis implications among patients infested with *S mansoni*. Because *Schistosoma* may be associated with colorectal carcinogenesis, it is necessary to create a specific protocol for screening of CRC in *Schistosoma*-endemic areas.^18^
